# Brain Neurotrophins and Plant Polyphenols: A Powerful Connection

**DOI:** 10.3390/molecules30122657

**Published:** 2025-06-19

**Authors:** Marco Fiore, Sergio Terracina, Giampiero Ferraguti

**Affiliations:** 1 Institute of Biochemistry and Cell Biology (IBBC-CNR), c/o Department of Sensory Organs, Sapienza University of Rome, 00185 Rome, Italy; 2 Department of Experimental Medicine, Sapienza University of Rome, 00185 Rome, Italy; sergio.terracina@uniroma1.it (S.T.);

**Keywords:** NGF, BDNF, polyphenols, Trk, addiction, curcumin, quercetin, resveratrol, hydroxytyrosol, EGCG, cocoa polyphenols

## Abstract

Neurodegenerative disorders, mental conditions, and cognitive decline represent significant challenges worldwide, with growing pieces of evidence implicating alterations in neurotrophin signaling as central to these diseases. Neurotrophins—such as nerve growth factor (NGF) and brain-derived neurotrophic factor (BDNF)—are indispensable for neuronal survival, differentiation, and synaptic plasticity, and their dysregulation is closely associated with various neuropathological situations. Similarly, dietary plant polyphenols, abundant in vegetables, fruits, wine, tea, and extra virgin olive oil, show powerful anti-inflammatory, antioxidant, and anti-apoptotic activities. This narrative review critically addresses the evolving body of evidence that links plant polyphenols and brain neurotrophins, emphasizing several molecular mechanisms by which polyphenols regulate and modulate neurotrophin signaling. Crucial pathways include mitigation of neuroinflammatory responses, activation of intracellular cascades such as the cAMP response element-binding protein (CREB), epigenetic modulation, and the diminution of oxidative stress. Together, these effects contribute to potentiated enhanced synaptic function, neuronal integrity, and better learning and memory processes. Moreover, this narrative review examines how polyphenol-induced upregulation of neurotrophins may alleviate conditions associated not only with neurodegeneration but also with addiction and mood disorders, suggesting extensive therapeutic approaches. Findings from clinical investigations and animal models are presented to sustain the neuroprotective role of polyphenol-rich diets. Lastly, future research directions are recommended, focusing on polyphenol bioavailability optimization, considering combinatory dietary stratagems, and proposing personalized nutritional interventions. This wide-ranging perspective highlights plant polyphenols as encouraging modulators of neurotrophin pathways and supports their inclusion in approaches aimed at promoting brain health and counteracting neurodegenerative decline.

## 1. Introduction

Neurodegenerative diseases, mental disorders, and cognitive decline are among the most persistent health problems all over the world. These disorders not only affect millions of individuals worldwide but also place a significant affliction on caregivers and healthcare systems [1,2,3,4,5,6,7,8,9,10,11,12]. Brain health is influenced by multiple factors, including lifestyle choices, environmental exposures, and genetic predisposition [13,14,15,16,17,18,19,20,21,22,23,24]. Among the endogenous factors that regulate neuronal differentiation, survival, and synaptic plasticity, neurotrophins play a pivotal role [25,26,27,28,29,30,31,32,33,34,35,36]. These peptides, including nerve growth factor (NGF), brain-derived neurotrophic factor (BDNF), and others, are crucial for maintaining cognitive function and protecting against neurodegeneration [37,38,39]. They sustain the growth, survival, and maintenance of neuronal cells, and their dysregulation has been associated with various neurological conditions [26,40,41,42,43,44,45,46].

Similarly, dietary components, especially plant-derived polyphenols, have gathered important interest due to their putative neuroprotective effects [47,48,49,50,51,52,53,54,55,56]. Polyphenols, which are abundant in vegetables, tea, wine, fruits, and other plant-based foods, display a wide range of biological activities. These include antioxidant, anti-inflammatory, and anti-apoptotic properties, which contribute to their protective effects on brain health [57,58,59,60,61,62,63,64]. Pieces of evidence suggest that polyphenols regulate neurotrophin signaling, thus influencing brain functioning and opposition against neurodegenerative disorders. This regulation arises through several mechanisms, such as activating signaling pathways, enhancing neurotrophin expression, and protecting neurons from oxidative stress and inflammation [65,66,67].

This narrative review aims to explore the connection between brain neurotrophins and plant polyphenols, offering insights into their combined potential for neuroprotection and mental and learning improvements. The understanding of the mechanisms by which neurotrophins and polyphenols cooperate is crucial for developing novel therapeutic approaches. By examining their roles individually and in combination, we can disclose valuable understandings of how dietary interventions may support brain health. This narrative review aims to present a wide-ranging overview of plant polyphenols, and neurotrophins and their synergistic effects on neuroprotection and cognitive function.

Furthermore, the potential therapeutic applications of neurotrophins and polyphenols extend beyond neurodegenerative disorders. Recent studies have highlighted their roles in mental health, suggesting that they may alleviate symptoms of anxiety, depression, and other mood disorders. The interaction between neurotrophins and polyphenols may also play a role in addiction, where neuroplasticity and reward pathways are significantly connected. By exploring these associations, we can better recognize how dietary and lifestyle interventions may participate in mental well-being and addiction recovery.

## 2. Materials and Methods

In March 2025, a selected literature search was conducted to identify important papers across multiple databases, including PubMed, Scopus, and Web of Science (WOS), to sustain this narrative review. Articles were selected using keywords such as “brain”, “polyphenols”, “neurotrophins”, “animal models”, and “human”, without restriction on publication year. Restricted inclusion criteria were as follows: (1) English-language articles, (2) original studies on brain neurotrophins and polyphenols, and between polyphenols we considered in the discussion resveratrol, epigallocatechin gallate (EGCG), quercetin, curcumin, hydroxytyrosol/oleuropein/tyrosol (olive polyphenols), cocoa polyphenols. Letters, editorials, and case reports were included where appropriate. Studies meeting these criteria were further analyzed, and relevant data were extracted from each paper.

## 3. Neurotrophins

Neurotrophins are a family of growth factors essential for the development, survival, and function of neurons [68,69,70,71,72,73,74]. The principal neurotrophins include nerve growth factor (NGF), brain-derived neurotrophic factor (BDNF), neurotrophin-3 (NT-3), and neurotrophin-4/5 (NT-4/5) [36,75,76,77,78,79]. These peptides are important for maintaining the health and functionality of the nervous system. Among these, NGF and BDNF are particularly vital for synaptic plasticity, memory, and learning [69,80,81,82]. The dysregulation of neurotrophin signaling has been associated with various neurological and psychiatric disorders, including Parkinson’s disease, Alzheimer’s disease, and depression [26,83,84,85,86,87,88].

The action of neurotrophins is based on two classes of receptors: tropomyosin receptor kinase (Trk) receptors and the p75 neurotrophin receptor (p75NTR) [89,90,91,92,93]. Trk receptors, including TrkA, TrkB, and TrkC, primarily mediate survival and differentiation signals [93,94,95,96,97]. For instance, TrkA binds NGF, TrkB binds BDNF and NT-4/5, and TrkC binds NT-3. These bonds activate intracellular signaling pathways that promote neuronal growth, survival, and synaptic plasticity. On the other hand, p75NTR can induce apoptosis under certain conditions, particularly when neurotrophin levels are low or when the receptor is unbound [98,99,100,101,102,103]. This Janus role of neurotrophin receptors underlines the complexity of neurotrophin signaling in brain health.

According to their fundamental role in brain health, approaches aimed at modulating neurotrophin levels and action are of intriguing interest in neuroscience research. Indeed, therapeutic approaches may include the use of peptides, small molecules, and gene therapy to enhance neurotrophin signaling [104,105,106,107,108,109,110]. Furthermore, lifestyle interventions such as dietary modifications and physical exercise have been shown to influence neurotrophin levels, offering non-pharmacological avenues for promoting brain health [111,112,113,114,115].

NGF and BDNF, in particular, have been widely studied due to their role in neurogenesis and synaptic plasticity [75,94,116,117,118,119]. NGF and BDNF are highly expressed in the hippocampus, cortex, and basal forebrain—regions associated with cognitive function and memory [120,121,122,123,124,125,126]. NGF and BDNF expression are regulated by several factors, including physical activity, stress, and diet [127,128,129,130,131]. For example, exercise has been shown to modulate NGF and BDNF levels, which are associated with improved cognitive function and reduced risk of neurodegenerative diseases [132,133,134]. Stress, on the other hand, can alter NGF and BDNF levels, resulting in cognitive impairment and mood disorders [69,111,135,136]. Significantly, emerging pieces of evidence indicate that dietary polyphenols can modulate NGF and BDNF levels, providing a promising opportunity for cognitive enhancement and neuroprotection [65,137,138,139,140,141].

Previous studies have indicated that NGF and BDNF expression declines with age [142,143,144,145], a feature associated with cognitive disruption and increased susceptibility to neurodegenerative conditions. The molecular mechanisms underlying NGF and BDNF modulation include transcriptional control via CREB (cAMP response element-binding protein) [146,147,148,149] and connections with neuroinflammatory processes [67,150,151]. Indeed, CREB is a transcription factor that binds to the promoter region of the BDNF gene, enhancing its expression [147,152]. Neuroinflammation, which is quite common in both aging and neurodegenerative disorders, can negatively affect NGF and BDNF signaling, further potentiating cognitive decline [153,154,155,156,157]. Since NGF and BDNF play such a key role in neuronal survival, interventions aimed at modulating their levels, either pharmacologically or dietary interventions, are a focus of current research.

Moreover, animal model studies have shown that genetic knockdown of BDNF leads to severe cognitive deficits, supporting its essential role in brain function [158,159,160,161,162,163,164,165]. By contrast, the upregulation of NGF and BDNF through exercise or dietary modifications has been associated with potentiated synaptic plasticity, suggesting potential therapeutic strategies for neurodegenerative conditions [112,113,114,115]. Recent clinical trials exploring the use of BDNF enhancers highlight promising directions in neuromodulation and cognitive therapy [166,167,168,169,170]. Indeed, these trials aimed to disclose safe and effective procedures for boosting neurotrophin levels in humans, with the purpose of improving cognition and reducing the risk of neurodegenerative disorders.

## 4. Plant Polyphenols

Polyphenols are bioactive compounds found in vegetables, fruits, coffee, tea, wine, and various medicinal plants [54,171,172,173,174,175,176]. They are classified into different categories, including phenolic acids, flavonoids, lignans, and stilbenes, each showing distinctive biological activities [173,177,178,179]. Flavonoids, particularly anthocyanins, catechins, and quercetin, are among the most investigated polyphenols in the context of brain health [180,181]. These chemicals are known for their powerful antioxidant properties [173,176,182,183,184], which support the neutralization of free radicals and counteract oxidative stress—a crucial factor in neurodegeneration.

**Figure 1:**
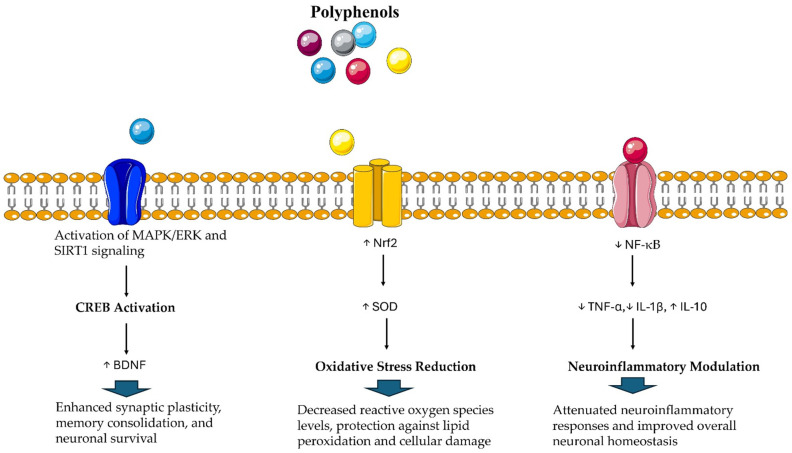
Through distinct biomolecular pathways, polyphenols exert a variety of health-promoting effects, contributing to neuroprotection, antioxidant defense, and the modulation of neuroinflammatory processes. Servier Medical Art by Servier is licensed under a Creative Commons Attribution 3.0 Unported License (https://creativecommons.org/licenses/by/3.0/ accessed on 18 May 2025). ↑ Indicates elevation. ↓ Indicates reduction.

Polyphenols possess multiple beneficial effects, including neuroprotective antioxidant, and anti-inflammatory properties (Figure 1). They regulate key signaling pathways involved in mitochondrial function, oxidative stress, and neuronal survival [185,186,187,188,189]. For instance, polyphenols can modulate the Nrf2 pathway, which improves the expression of antioxidant enzymes and protects neurons from oxidative damage [190,191,192].

Furthermore, polyphenols can constrain the NF-κB pathway, decreasing inflammation and promoting neuronal health [141,190,192,193]. Particularly, polyphenols can cross the blood–brain barrier (BBB), permitting a direct communication between neuronal circuits and molecular targets within the brain [54,194,195]. This aptitude to breach the BBB is critical for its neuroprotective effects, as it allows polyphenols to act straightforwardly within the central nervous system (Table 1).

Among the most potent neuroprotective polyphenols are resveratrol, hydroxytyrosol, curcumin, EGCG, and quercetin. These chemicals have been shown to potentiate neuronal function through several mechanisms, including the activation of the neurotrophic signaling pathways [65,196,197]. Resveratrol, a stilbene commonly found in grapes, red wine, and other plants has been described to modulate synaptic plasticity and Sirtuin 1 (SIRT1) (see Table 1), a longevity-associated protein that interacts with BDNF signaling pathways [141,198,199,200,201]. Indeed, SIRT1 activation by resveratrol can potentiate BDNF expression, promoting cognitive function and neuronal survival [202,203,204,205,206]. Curcumin, a bioactive chemical in turmeric, has revealed neuroprotective effects in Alzheimer’s animal models by reducing amyloid-beta plaque deposition and elevating BDNF levels [207,208,209]. Curcumin’s anti-inflammatory properties also participate to its neuroprotective actions, since chronic inflammation is a characteristic of neurodegenerative diseases [207,208,209]. EGCG, found in green tea, has been associated with reduced neuroinflammation and enhanced neurogenesis, eliciting improved cognitive function [210,211,212,213,214]. Indeed, EGCG also regulates mitochondrial function reducing oxidative stress, and enhancing energy production in neurons [215,216,217,218].

Quercetin, a flavonoid present in many vegetables and fruits, has been shown to protect neurons from apoptosis and oxidative stress [215,216,217,218]. Indeed, quercetin regulates signaling pathways such as mitogen-activated protein kinase (MAPK) and PI3K/Akt [219,220] (see Table 1), which are involved in cell survival and neuroprotection. The ability of quercetin to boost mitochondrial action and reduce neuroinflammation further emphasizes its putative function in brain health.

A parallel dissertation can be told for hydroxytyrosol. Indeed, hydroxytyrosol and oleuropein are well known for their antioxidant, anti-inflammatory, and neuroprotective properties leading to the counteraction of neurodegenerative diseases of the central/peripheral nervous system, improving adult neurogenesis, senescence, and lifespan [221,222,223,224].

**Figure 2:**
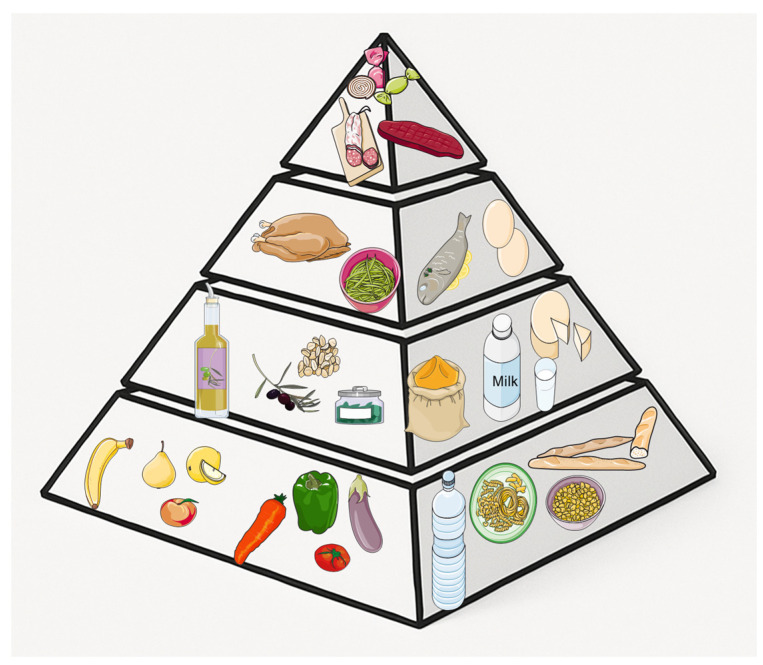
The Mediterranean diet is rich in polyphenols: fruits and vegetables, in particular berries, red grapes and red wine, green and black tea, cocoa, chocolate, olives and extra virgin olive oil. It is suggested to eat at least 2 portions of vegetables in the main meals with 1–2 portions of fruits and cereals. Nuts, seeds and olives (1–2 servings), but also milk and dairy products (2–3 servings), herbs and spices and olive oil (3–4 servings) should be consumed daily. Finally, weekly consumption of fish, crustaceans and mollusks (at least 2 servings), poultry (1–2 servings), eggs (2–4 servings) and legumes (at least 2 servings) is indicated. Meat, sweets, and cold cuts should be eaten sparingly (no more than 1 time a week). Water should be the main drink, but moderate consumption of wine is permitted. Servier Medical Art by Servier is licensed under a Creative Commons Attribution 3.0 Unported License (https://creativecommons.org/licenses/by/3.0/ accessed on 18 May 2025).

In addition to specific polyphenols, dietary applications rich in polyphenols, such as traditional Asian diets and the Mediterranean diet, have been related to enriched cognitive functioning and reduced incidence of neurodegenerative conditions [225,226,227,228,229]. The Mediterranean diet, which embraces high consumption of vegetables, fruits, seeds, nuts, and extra virgin olive oil, is particularly rich in polyphenols (Figure 2).

Studies have disclosed that adherence to the Mediterranean diet is associated with better cognitive function and a lower risk of brain aging [230,231,232,233,234]. Similarly, traditional Asian diets, which involve soy products, green tea, and several herbs, offer a rich source of polyphenols that participate to brain health [231,232,235].

Clinical studies indicate that long-term consumption of polyphenol-rich foods contributes to improved neuronal resilience and better brain aging against stress-related damage. For example, studies on elderly individuals disclosed that regular consumption of polyphenol-rich foods was associated with improved cognitive performance and a lower risk of cognitive decline [166,236,237,238,239]. These conclusions highlight the importance of dietary polyphenols in preventing neurodegenerative diseases and maintaining brain health.

## 5. Polyphenols and the Gut–Brain Axis

Polyphenols may also have a role in regulating gut–brain interactions [240,241,242,243]. Indeed, the gut microbiota can absorb polyphenols into bioactive metabolites that can cross the blood–brain barrier, influencing brain function. For example, polyphenols can promote the growth of beneficial gut bacteria that produce short-chain fatty acids (SCFAs), which have been shown to influence brain function and reduce inflammation [244,245].

Indeed, beyond their straight actions in the central nervous system (CNS), plant polyphenols greatly reshape the gut microbiota environment, modulating bioactive metabolites affecting brain functions [246]. In the colon, unabsorbed polyphenols undertake extensive modifications by resident bacteria into SCFAs and low-molecular-weight phenolic acids, which present enriched blood–brain barrier permeability and intestinal absorption. By acting as a sort of prebiotics, polyphenols selectively enrich SCFA-producing taxa (e.g., *Faecalibacterium* sp., *Roseburia* sp.) and beneficial bifidobacteria and lactobacilli while reducing pathobionts. This transformed microbiome not only elevates systemic levels of propionate, butyrate, and acetate but also could modulate circulating indole derivatives and tryptophan, important precursors for brain neurotrophins and neuromodulators [246].

As for SCFAs, they serve as molecular messengers of the microbiota–gut–brain axis by linking free-fatty-acid receptors on enteroendocrine cells and vagal afferents [247], and by acting on microglia throughout the SCFA–microglia pathway [247]. Indeed, butyrate acts as a histone deacetylase inhibitor in the CNS, leading to epigenetic inhibition of NGF/BDNF gene promoters in the hippocampus [247]. This elicits CREB activation and neurotrophin release, increasing synaptic plasticity and counteracting inflammation. Concomitantly, microbial phenolic metabolites, (i.e., urolithins and p-coumaric acid) might exert anti-inflammatory actions on gut-associated lymphoid tissue, reducing peripheral cytokine release that could compromise neurotrophin signaling within the blood–brain barrier [246].

Animal and human data highlight that polyphenols-rich long-term dietary patterns (e.g., Mediterranean, plant-forward diets) correlate with higher plasma and CSF levels of BDNF, SCFA-enriched microbiota, and better cognitive performance during aging [241,248]. Thus, the integration of prebiotic modulation with potentiated microbial populations could elicit the release of neuroactive small molecules, and epigenetic upregulation of neurotrophin pathways to further underscore the subtle polyphenols’ role in a multidimensional gut–brain axis mechanism [240,241,242,243].

## 6. Brain and Plant Polyphenols

Recent research focuses on the aptitude of plant polyphenols to potentiate neurotrophin signaling and expression. Many studies show that polyphenol-rich diets may increase NGF and BDNF levels, promoting cognitive function and brain cells’ resilience. Several biomolecular mechanisms support this feature:

### 6.1. Epigenetic Modulation

Polyphenols can modify gene expression through DNA methylation and histone modification [249,250,251,252,253,254], modulating neurotrophin synthesis and release. For example, curcumin and resveratrol have been shown to affect histone acetylation, leading to elevated expression of BDNF [255,256,257,258]. These epigenetic modifications can have long-lasting effects on brain function and neuronal plasticity. Furthermore, polyphenols can influence DNA methylation configurations [259,260,261], which play a subtle role in modulating gene expression. Thus, by regulating these epigenetic mechanisms, polyphenols can increase the synthesis of neurotrophins and promote neuronal health.

### 6.2. Activation of Cellular Pathways

Polyphenols trigger pathways such as cAMP response element-binding protein (CREB), which is crucial for the transcription of NGF and BDNF [138,262]. CREB is a key transcription factor that, when activated, binds to the promoter region of the BDNF gene, potentiating its expression [263,264]. Quercetin and EGCG have been shown to activate CREB through various signaling cascades, including the MAPK/ERK and PI3K/Akt pathways [265,266] (see Table 1). These pathways play a role in cell differentiation, survival, and synaptic plasticity, emphasizing the multifaceted role of polyphenols in brain functions.

### 6.3. Mitigation of Neuroinflammation

By dropping inflammatory cytokines, polyphenols may prevent neurotrophin deprivation and improve their protective effects [176,267,268,269,270]. Chronic inflammation is a crucial factor in neurodegenerative conditions, and polyphenols can prevent the synthesis and release of pro-inflammatory cytokines such as TNF-α and IL-1β [271,272,273,274,275] (see Table 1). This anti-inflammatory achievement supports preserving neurotrophin levels, inducing neuronal health. Furthermore, polyphenols can modulate the activity of microglia [47,276,277], the brain’s resident immune cells, decreasing their pro-inflammatory reactions and stimulating a neuroprotective environment.

### 6.4. Oxidative Stress Reduction

Polyphenols may reduce oxidative stress [239,276,278,279], maintaining neuronal integrity and supporting the neurotrophin role. Indeed, oxidative and nitrosative stresses are major contributors to neuronal damage and neurodegeneration. Polyphenols, throughout their solid antioxidant properties, can counteract free radicals and modulate endogenous antioxidant defenses, thus protecting neurons and eliciting neurotrophin signaling. For example, polyphenols can enhance the activity of antioxidant enzymes such as superoxide dismutase (SOD) and catalase [280,281,282], which have a key role in sustaining cellular redox balance.

### 6.5. Model Studies

Studies on animal models and human subjects support the role of polyphenols in cognitive enhancement. For example, flavonoid-rich diets have been associated with improved memory and learning capabilities [283,284]. In animal models, polyphenols supplementation such as cocoa, green tea, and blueberries has been shown to potentiate cognition and elevate hippocampal BDNF [285,286,287,288]. These findings indicate that dietary polyphenols may modulate brain resilience and function by enhancing synaptic plasticity, which is crucial for learning and memory processes.

Furthermore, several studies have suggested that people consuming polyphenol-rich diets (such as fruits, vegetables, and tea) demonstrate a reduced risk of neurodegenerative disorders [289,290,291]. These observational investigations provide convincing evidence for the protective effects of polyphenols on brain health, also through their effects on the gut–brain axis [244,246,292].

Clinical trials have provided additional indications of the neuroprotective effects of polyphenols. A previous study on elderly people consuming a Mediterranean diet rich in polyphenols revealed enriched cognitive performance and elevated plasma BDNF [293]. Additionally, randomized controlled trials on polyphenol supplementation have reported benefits in delaying cognitive decline in patients with mild cognitive impairment [139,294,295,296]. Indeed, supplementation with curcumin, resveratrol, or EGCG improved cognitive tasks and elevated BDNF levels in clinical individuals [139,294,295,296] (Table 2).

As for cocoa polyphenols, a randomized, double-blind, parallel-group study on 60 healthy volunteers between 50 and 75 years old who consumed a cocoa powder, a red berries mixture or a combination of both for 12 weeks it was shown an improvement in executive function without significant difference in BDNF and NGF-receptor sera levels [297]. In an Alzheimer in vitro study, cocoa powder triggers neuroprotective and preventive effects by modulating BDNF signaling pathway [298], whereas a diet enriched with high-phenolic cocoa potentiates hippocampal brain-derived neurotrophic factor expression and neurogenesis in healthy adult mice with subtle effects on memory [285].

Another study examined the effects of dark chocolate intake on improving brain function during cognitive tasks using functional magnetic resonance imaging (fMRI) [238]. In this randomized, single-blinded, crossover, and dose-comparison study, 26 healthy middle-aged participants ingested dark chocolate (25 g) either with a low concentration (LC) (211.7 mg) or a high concentration (HC) (635 mg) of cacao polyphenols (mainly epicatechin and theobromine) [238]. Thereafter, their brain activities were analyzed during continuous and effortful cognitive tasks relevant to executive functioning using fMRI in two consecutive 15 min sessions (25 and 50 min after ingestion) [238]. The authors observed significant interaction effects between chocolate consumption and brain activity measurement sessions in the left dorsolateral prefrontal cortex and left inferior parietal lobule [238].

## 7. Polyphenols, Neurotrophins, and Addiction

Addiction is a multifaceted disorder involving dysregulation of neurochemical pathways, particularly those associated with neuroplasticity and reward processing [299,300,301]. Chronic abuse of addictive substances such as nicotine, alcohol, and drugs alters neurotrophin levels, particularly NGF and BDNF [302]. The dysregulation of NGF and BDNF and other neurotrophins contributes to the neuroadaptive changes, including alterations in synaptic connectivity and strength in brain areas involved in motivation and reward [69,303,304].

Plant polyphenols have been studied for their putative role in mitigating addiction-related neuroadaptations. Indeed, several studies, mostly in animal model studies, suggest that polyphenols can modulate neurotransmitter signaling and neurotrophin presence, which both have a critical role in the brain’s reward structure. For example:

### 7.1. Resveratrol

Resveratrol has been shown to reduce drug-seeking behavior by enhancing BDNF levels and modulating dopamine receptor expression in addiction-related brain regions [305,306,307]. Indeed, resveratrol’s capability to modulate the dopaminergic system is particularly significant [308,309,310], as dopamine has a key role in the reward circuitry and the reinforcing effects of abuse substances. Further, in a mouse model, the consumption of resveratrol through metabolite formation may have a protective role by reducing free radical presence and tempering the BDNF involved in hepatic disruption induced by chronic alcohol abuse [197,311,312].

### 7.2. Curcumin

Curcumin shows neuroprotective actions in addiction models by dropping inflammation and oxidative stress, which participate in relapse vulnerability [313,314,315,316]. Indeed, chronic exposure to substance abuse often leads to elevated neuroinflammation and oxidative stress, which can harm neurons and damage cognitive function. Thus, curcumin’s anti-inflammatory and antioxidant properties could mitigate these effects, supporting neuronal health and reducing the possibility of relapse. Curcumin has been also shown to regulate neurotransmitter systems, including dopamine and serotonin, which are both involved in abuse dependence and mood modulation [317,318,319].

### 7.3. Green Tea Polyphenols

Green tea polyphenols, particularly EGCG, have demonstrated the ability to modulate withdrawal symptoms [320] and normalize neurotrophin expression [321,322,323,324,325]. Indeed, withdrawal from abuse substances can lead to a variety of outcomes, including depression, anxiety, and cognitive changes. EGCG’s neuroprotective actions could alleviate these symptoms by potentiating neurotrophin signaling and stimulating neuronal recovery. Furthermore, EGCG has been shown to regulate the activity of the dopamine system [326,327], which has a key role in the rewarding drug effects [328,329].

### 7.4. Olive Polyphenols

Oleuropein, tyrosol, and hydroxytyrosol, obtained from the olive plant leaves and seeds, have also shown potential in substance abuse research. Hydroxytyrosol and tyrosol, found in olive oil and extra virgin olive oil, show strong anti-inflammatory and antioxidant abilities [330]. These polyphenols are known to protect neurons from oxidative stress [331,332], enhance neurotrophin signaling [65,137,197,333], and potentially modulate the neurotoxic effects of addictive substances [334]. Oleuropein, another olive polyphenol, shows neuroprotective actions by regulating dopamine signaling and reducing neuroinflammation [335,336,337,338,339]. Quite interestingly, hydroxytyrosol is also known as DOPET (3,4-dihydroxy-phenylethanol), a well-known dopamine metabolite, and is known to be endogenously secreted and present in mammal body fluids at low concentrations [340,341], further supporting the key role of hydroxytyrosol in the addiction/reward systems.

## 8. Discussion and Conclusions

The association between plant polyphenols and neurotrophins offers a promising opportunity for neuroprotection and cognitive potentiation. Based on the increasing prevalence of neurodegenerative diseases associated with aging and cognitive deterioration, modifications in dietary approaches could enhance neurotrophin activity offering non-invasive accessible interventions. These strategies are particularly attractive since they can be easily incorporated into daily life and have the potential to boost brain health without the necessity for pharmaceutical interventions.

Future research should focus on clarifying the detailed molecular mechanisms through which polyphenols modulate neurotrophin signaling. The comprehension of these mechanisms will be crucial for developing directed dietary interventions and therapeutic strategies. For instance, disclosing specific polyphenols that most successfully potentiate neurotrophin signaling and expression might lead to the development of supplements or functional foods intended to sustain brain health. Of course, additional research should explore how polyphenols interact with other dietary components and lifestyle factors that might influence neurotrophin activity.

Clinical investigations are necessary to establish optimal dosages, bioavailability, and long-term effects of polyphenol-rich diets. These studies should aim to determine international evidence-based guidelines for polyphenol consumption to optimize their neuroprotective benefits. Indeed, factors such as genetic background, age, sex, and pre-existing health conditions could have a role in the effectiveness of polyphenol interventions, and personalized approaches could be necessary to obtain the best outcomes. Moreover, long-term studies are indispensable to assess the safety and sustainability of polyphenol-rich diets over extended times.

It should also be noted that investigating the cooperative interactions between different polyphenols and other bioactive compounds could improve their neuroprotective ability. For instance, combining polyphenols with vitamins, omega-3 fatty acids, or minerals could elicit additive or synergistic outcomes that further sustain brain health [342,343,344]. Exploring this synergistic supplementation could disclose comprehensive dietary strategies that might raise cognitive function and protect against neurodegenerative disorders.

The ability of polyphenols to regulate neurotrophins is not static but differs noticeably with both the dose and duration of supplementation. Acute interventions often induce prompt, transient spikes in circulating and central neurotrophin levels, while chronic schedules are required to maintain and strengthen these modifications.

In healthy volunteers, *acute* doses of cocoa flavanols elicited a rise in serum BDNF [345,346]. Similarly, EGCG in mice increased hippocampal BDNF mRNA [347]. Long-lasting supplementation (4–12 weeks) is often required to achieve durable neurotrophic upregulation: in older adults, daily consumption of circa 500 mg cocoa flavanols for 12 weeks raised resting plasma BDNF and improved performance on memory tasks [348]. Rodent studies likewise show that resveratrol elevates hippocampal BDNF expression [349] and modulates dendritic spine density [350], whereas a single dose has debated effects. These observations emphasize the necessity for cautious titration of polyphenol assumption to balance pro-neurotrophic signaling against metabolic clearance and/or potential pro-oxidant effects at supraphysiological concentrations [351,352].

However, although several papers evidenced the effects of polyphenols on neurotrophin synthesis and release, some clinical trials in aged people failed to disclose significant changes in circulating neurotrophins following polyphenol administration [139]. For instance, 12 weeks of supplementation with cocoa flavanols in cognitively healthy elderly subjects found no modifications in plasma BDNF levels versus the placebo group [297]. Similarly, it was observed that curcumin administration in amateur long-distance runners did not significantly change serum BDNF [294]. Such inconsistencies probably suggest heterogeneity in polyphenol source, assay sensitivity, duration, dose, and baseline nutritional and health status emphasizing the need for larger trials.

Despite the promising discoveries, several unresolved questions remain. The polyphenols bioavailability differs significantly depending on their chemical structure, concentration, and metabolic pathways. Polyphenols may be metabolized quickly or may have limited absorption in the gastrointestinal tract, decreasing their efficacy. According to this issue, approaches to improve their absorption and stability, such as dietary co-administration and nanoencapsulation, should be investigated [353,354,355,356,357]. To improve polyphenol stability, encapsulating polyphenols in nanoparticles could be considered to reduce degradation and improve their absorption too. Co-administration with other dietary elements, such as proteins or fats, could also enhance polyphenol bioavailability. For instance, curcumin displays important challenges due to its poor aqueous solubility (<0.1 mg/mL), low oral bioavailability (often below 1%), and rapid metabolism into sulfate and glucuronide conjugates, leading to low blood–brain barrier penetration [358,359,360,361]. These factors limit its therapeutic potential, particularly with oral administration. However, these limits have encouraged research into approaches aimed to improve curcumin’s bioavailability, including methods such as liposomes, nanoparticles, or co-administration with piperine (found in black pepper, that may inhibit glucuronidation and increase curcumin’s bioavailability) [362,363].

As for the Janus face of microglia and macrophages in neuroinflammation, it should be noted that microglia and infiltrating blood-derived macrophages show amazing phenotypic plasticity, swinging between neuroprotective and neurotoxic states depending on local signs. In their classic (“M2-like”) mode, they remove debris, release anti-inflammatory cytokines (e.g., TGF-β, IL-10) and may produce BDNF and NGF to sustain neuronal survival and synaptic remodeling [364,365,366]. On the contrary, chronic exposure to danger-associated molecular circuits or pro-inflammatory cues, they adopt a classic “M1-like” profile [367], releasing IL-1β, TNF-α, reactive oxygen species and nitric oxide that can disrupt neurotrophin signaling and aggravate neuronal damage [365,368].

Transcriptomics investigations disclosed a wide spectrum of intermediate activation conditions beyond this dualistic classification [369,370], including disease-associated microglia that could firstly constrain pathology but, under certain circumstances, participate in synaptic loss and demyelination [368]. Thus, the comprehension of how plant polyphenols regulate this delicate equilibrium, shifting microglia/macrophages toward anti-inflammatory, neurotrophin-releasing phenotypes, will be significant for disclosing their complete therapeutic potential in neuropsychiatric and neurodegenerative conditions.

A descriptive study on the influence of polyphenol supplementation and exercise on depression and brain function parameters examined the combined antidepressant effects of exercise and polyphenol supplementation, with a special focus on specific polyphenolic chemicals such as curcumin, crocin, and quercetin, as well as different methods of physical exercise, including resistance and aerobic training [371]. Indeed, these interventions modulate cognitive function, depressive-like behaviors, and neurochemical biomarkers in animal models and humans [371]. The findings disclosed that both polyphenols and exercise independently participate in reduced anxiety, mood enhancement, and improved cognitive function through mechanisms such as neurotransmitter modulation, neurogenesis, and anti-inflammatory effects [371]. Intriguingly, the combined interventions showed a synergistic effect, providing significant health benefits in decreasing symptoms of depression and anxiety, enhancing cognitive processes, and supporting overall mental well-being [371].

In conclusion, the relationship between plant polyphenols and neurotrophins embodies a compelling field of investigation with important implications for brain function and disease prevention. Diets rich in polyphenol-containing foods may act as an effective and practical strategy to sustain learning and memory functions, modulating neurodegenerative processes. Indeed, the potential benefits of polyphenols cover beyond neuroprotection, as they may also reduce anxiety, support mood, and improve overall mental well-being. Future research should decode these findings into clinical applications, paving the way for polyphenol-based neuroprotective interventions. By disclosing evidence-based guidelines and investigating novel delivery methods, we could exploit the influence of polyphenols in promoting brain health and preventing learning and memory decline.

Likewise, interdisciplinary collaborations could be indispensable to make advancements in this field. Investigators from clinical medicine, neuroscience, nutrition, and pharmacology should collaborate to propose comprehensive research that addresses the complex association between neurotrophins, brain health, and diet. Public health initiatives should also promote the understanding of the benefits of polyphenol-rich diets and support their acceptance as a crucial portion of a healthy lifestyle.

## Figures and Tables

**Table 1:** Proposed biomolecular mechanisms dealing with the multifactorial action of polyphenols. *CREB activation*: polyphenols such as resveratrol and epigallocatechin gallate (EGCG) could stimulate MAPK/ERK and SIRT1 signaling, which in turn phosphorylate CREB and upregulate neurotrophins. This induces synaptic plasticity and sustains learning and memory. *Oxidative stress reduction*: through the activation of the Nrf2 pathway, polyphenols might potentiate the expression of antioxidant factors (e.g., SOD, catalase) that alleviate reactive oxygen species, thus protecting brain cells. *Neuroinflammation modulation*: by reducing the NF-κB pathway, polyphenols could depress the secretion of pro-inflammatory cytokines (e.g., TNF-α, IL-1β), decreasing neuroinflammation and potentially limiting neurodegeneration.

Polyphenols	Proposed Mechanisms	Molecular Pathway/Targets	Biological Outcomes
Resveratrol, epigallocatechin gallate (EGCG), curcumin	CREB activation	- Activation of MAPK/ERK and SIRT1 signaling—Phosphorylation of CREB—Upregulation of BDNF and other neurotrophins	Enhanced synaptic plasticity, memory consolidation, and neuronal survival
EGCG, resveratrol, quercetin	Reduction of oxidative stress	- Direct free radical scavenging—Activation of Nrf2 pathway leading to increased expression of antioxidant enzymes (e.g., SOD, catalase)	Decreased reactive oxygen species levels, protection against lipid peroxidation and cellular damage
Curcumin, resveratrol, hydroxytyrosol (olive polyphenols)	Modulation of neuroinflammatory mediators	- Inhibition of NF-κB signaling—Reduced expression of pro-inflammatory cytokines (e.g., TNF-α, IL-1β, IL-6)—Modulation of microglial activity	Attenuated neuroinflammatory responses and improved overall neuronal homeostasis

**Table 2:** Polyphenol regulation of neurotrophin receptors and signaling pathways. This table presents an overview of how various polyphenols interact with neurotrophin receptors (TrkA, TrkB) and crucial molecular pathways involved in neuroprotection, synaptic plasticity, and neuronal survival. Resveratrol, epigallocatechin gallate (EGCG), curcumin, quercetin, and hydroxytyrosol have been shown to regulate neurotrophins such as brain-derived neurotrophic factor (BDNF) and nerve growth factor (NGF), affecting pathways including PI3K/Akt signaling, CREB phosphorylation, and NF-κB inhibition. These biomolecular processes participate in potentiated neuronal resilience, improved memory and learning processes, and a protective action against neurodegeneration. Experimental models, ranging from in vitro neuronal cultures to in vivo investigations on neurodegenerative conditions (Alzheimer’s, Parkinson’s, and addiction models), sustain the neurotrophic-potentiating actions of polyphenols. ↑ Indicates elevation.

Polyphenol	Targeted Neurotrophins/Receptors	Signaling Pathways Affected	Neurological Effects	Experimental Models
**Resveratrol**	BDNF, NGF, TrkB, TrkA	-↑ CREB phosphorylation—Activation of PI3K/Akt and ERK cascade	Improves memory, enhances synaptic plasticity	Rodent models, in vitro neuronal culture studies
**EGCG (green tea)**	BDNF, TrkB	-↑ BDNF expression—Suppression of NF-κB-driven inflammation	Protects neurons from oxidative stress, reduces apoptosis	Mouse models of cognitive dysfunction, aging studies
**Curcumin**	NGF, BDNF, TrkA, TrkB	- Modulates NF-κB—↑ Anti-apoptotic Bcl-2 pathways	Enhances neurogenesis, prevents inflammation	Parkinson’s and Alzheimer’s animal studies
**Quercetin**	BDNF, NGF	-↑ MAPK/ERK signaling—Regulates neurotrophin transcription factors	Enhances neuronal resilience, neuroprotection	In vitro hippocampal neuron models
**Hydroxytyrosol (olive polyphenols)**	NGF, BDNF, TrkA, TrkB	- Increases NGF synthesis—Regulates mitochondrial oxidative metabolism	Supports neuronal survival, counters neurodegeneration	Preclinical models of Alzheimer’s and aging disorders

## Data Availability

No new data were created or analyzed in this study. Data sharing is not applicable to this article.

## Abbreviations

The following abbreviations are used in this manuscript:

BBB	Blood–brain barrier
BDNF	Brain-derived neurotrophic factor
CREB	cAMP response element-binding protein
DOPET	3,4-dihydroxy-phenylethanol
EGCG	Epigallocatechin gallate
MAPK	Mitogen-activated protein kinase
NGF	Nerve growth factor
Nrf2	Nuclear factor erythroid 2-related factor 2
NT-3	Neurotrophin-3
NT-4/5	Neurotrophin-4/5
p75NTR	p75 neurotrophin receptor
SCFAs	Short-chain fatty acids
SIRT1	Sirtuin 1
SOD	Superoxide dismutase
Trk	Tropomyosin receptor kinase
WOS	Web of Science
